# Supplementary material for: Palladium Decorated, Amine Functionalized Ni-, Cd- and Co-Ferrite Nanospheres as Novel and Effective Catalysts for 2,4-Dinitrotoluene Hydrogenation
Source: Int J Mol Sci. 2022 Oct 30;23(21):13197. doi: 10.3390/ijms232113197 (PMC9657705; doi:10.3390/ijms232113197)
Supplement: Supplementary file 1 [file ijms-23-13197-s001.zip › ijms-1943254-supplementary.pdf]

Supporting Information

for

Palladium decorated, amine functionalized Ni-, Cd- and Co-ferrite nanospheres as novel and effective catalysts for 2,4-dinitrotoluene hydrogenation

Viktória Hajdu[1], Emőke Sikora[1], Ferenc Kristály[2], Gábor Muránszky[1], Béla Fiser[1], Béla Viskolcz[1], Miklós Nagy[1,*] and László Vanyorek[1,*]

[1]Institute of Chemistry, University of Miskolc, 3515 Miskolc-Egyetemváros, Miskolc, Hungary; nagy.miklos@uni-miskolc.hu (M.N.); kemvanyi@uni-miskolc.hu (L.V.)

[2]Institute of Mineralogy and Geology, University of Miskolc, 3515 Miskolc-Egyetemváros, Hungary

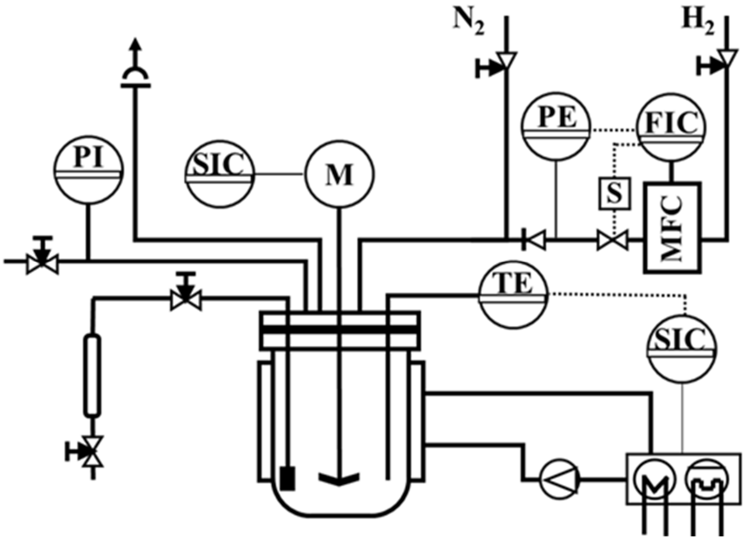

Figure S1: Piping and instrumentation diagram (P&ID) of the Büchi Uster Picoclave reactor system for catalytic hydrogenation tests.

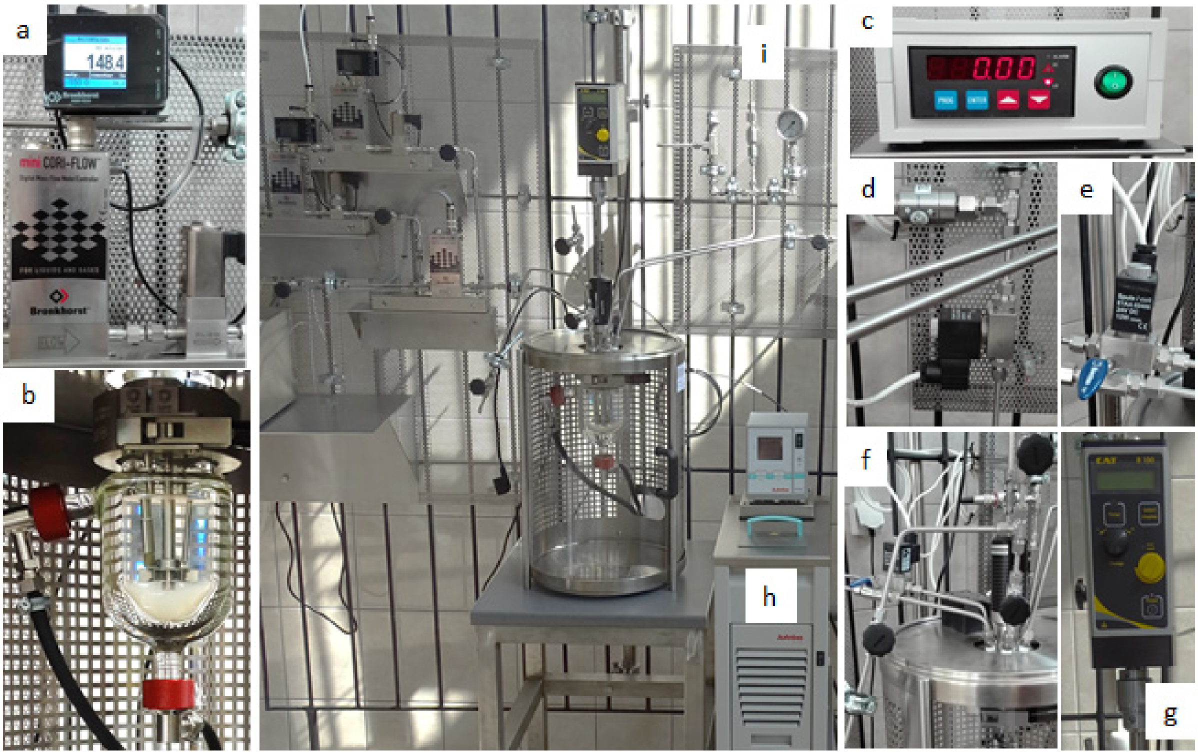

Figure S2: Büchi reactor system, and its parts: flowmeter (a), reactor vessel (b), pressure regulator (c), magnetic valves (d, e), sampling system (f), mixing motor (g), heating circulator (h) and safety equipment, rupture disc and relief valve (i).

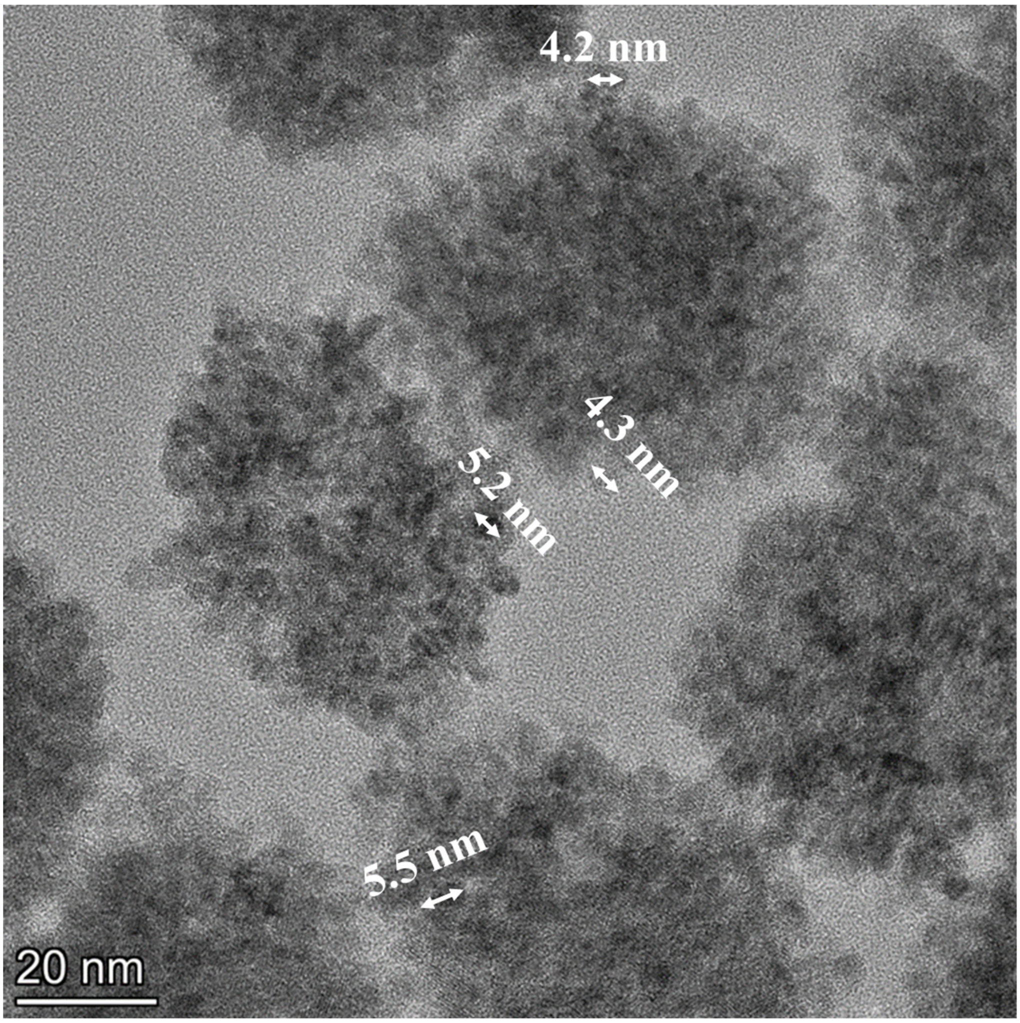

Figure S3: HRTEM picture of the $CoFe_2O_4$- NH_2 nanoparticles

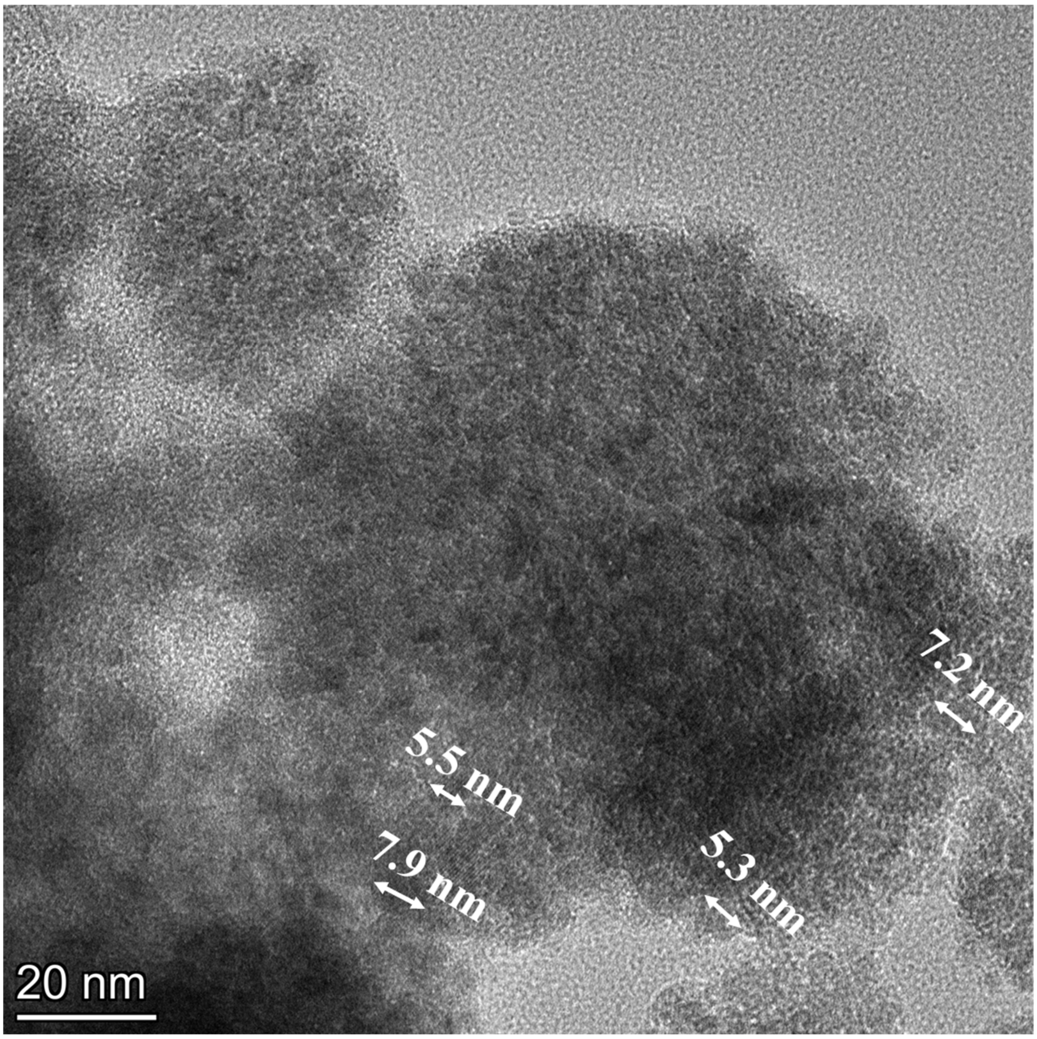

Figure S4: HRTEM picture of the $NiFe_2O_4$- NH_2 nanoparticles

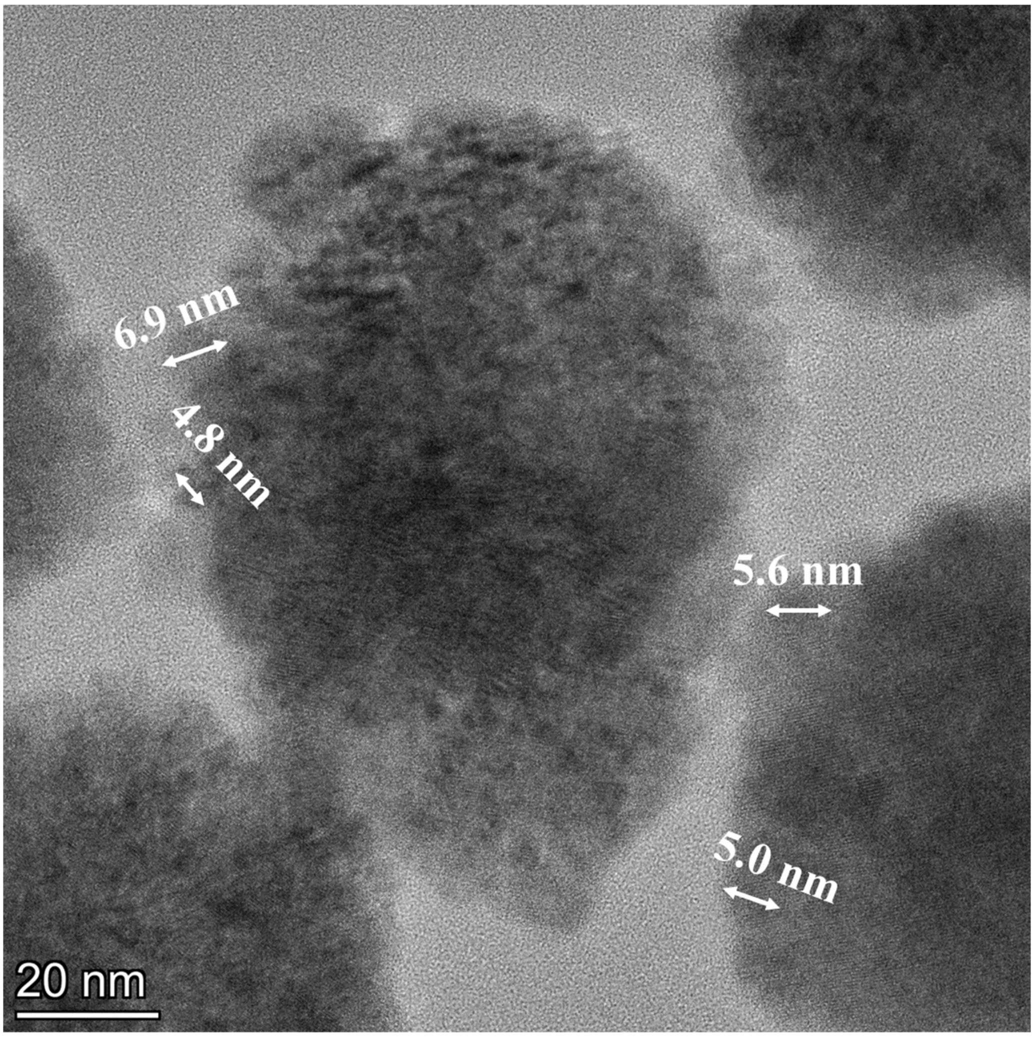

Figure S5: HRTEM picture of the $CdFe_2O_4$- NH_2 nanoparticles

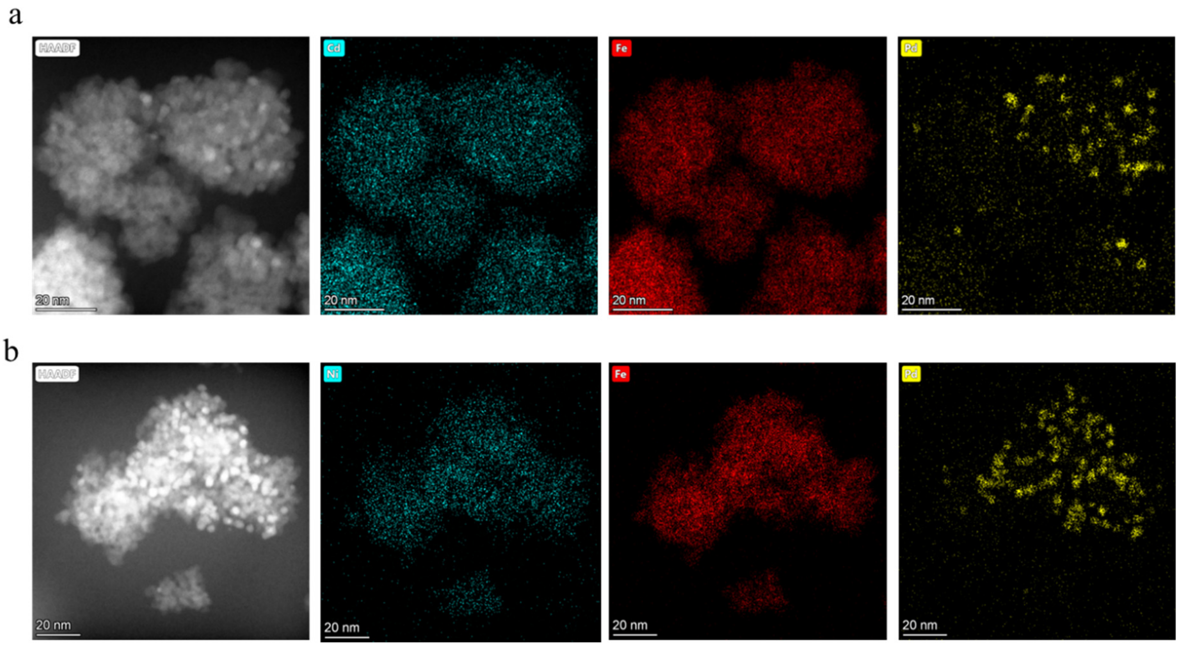

Figure S6: Element maps of the Pd/$CdFe_2O_4$-NH_2 (a) and Pd/$NiFe_2O_4$-NH_2 (b)

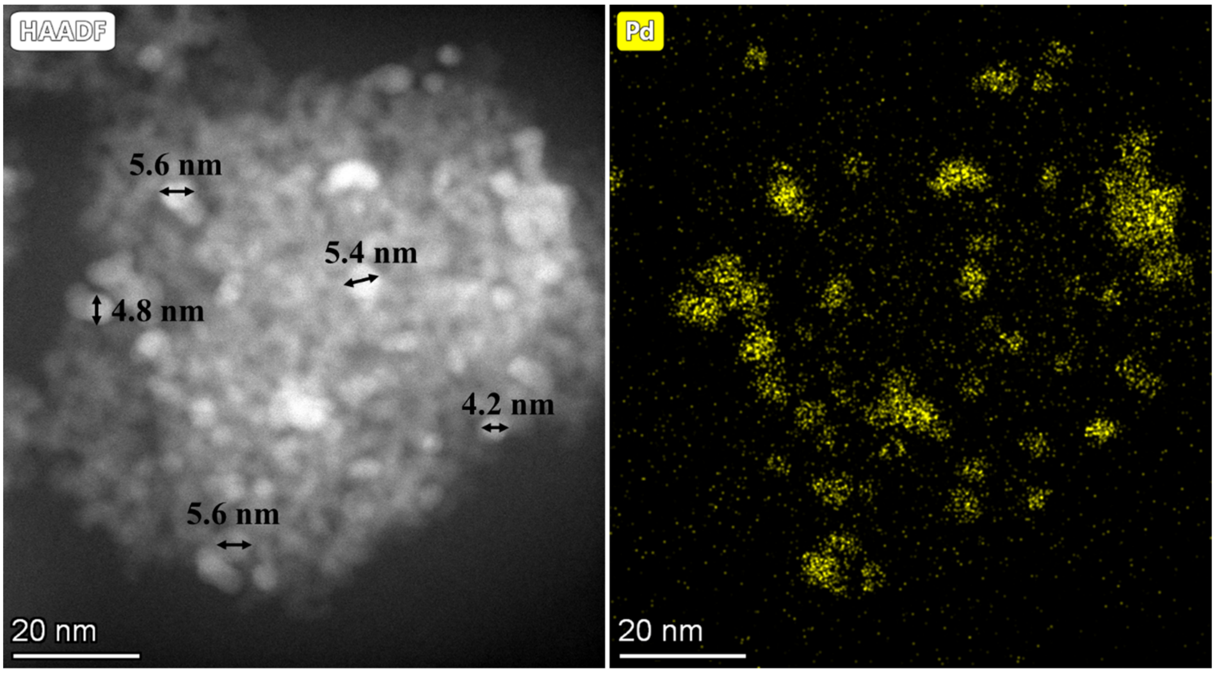

Figure S7: HAADF picture and element mapping of the Pd/$CoFe_2O_4$-NH_2 catalyst

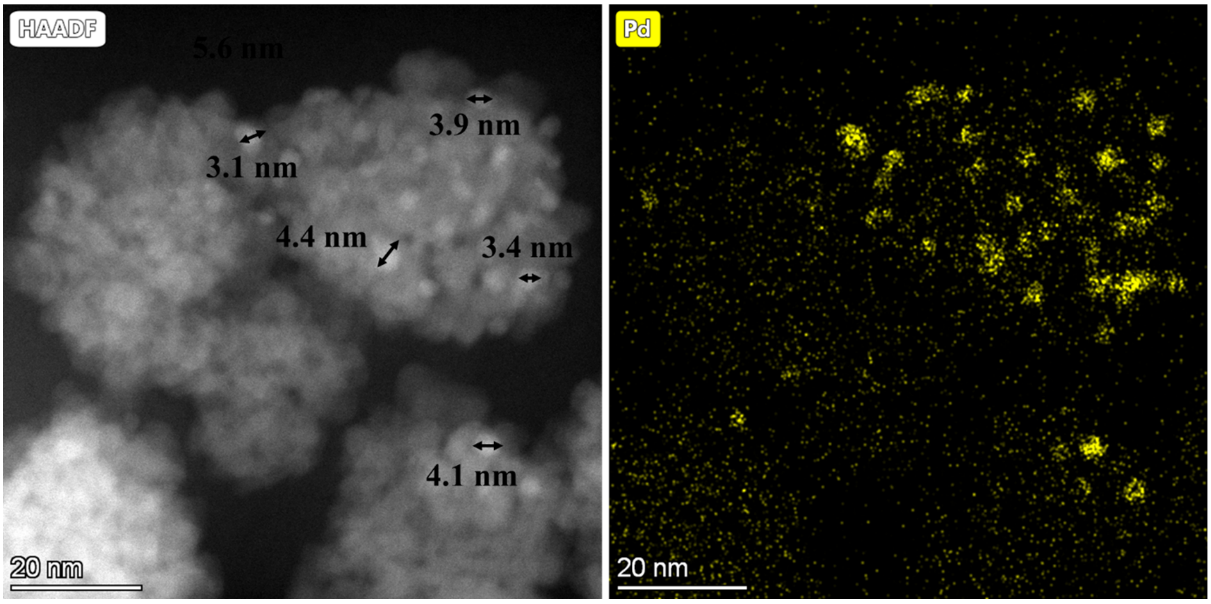

Figure S8: HAADF picture and element mapping of the Pd/$CdFe_2O_4$-NH_2 catalyst

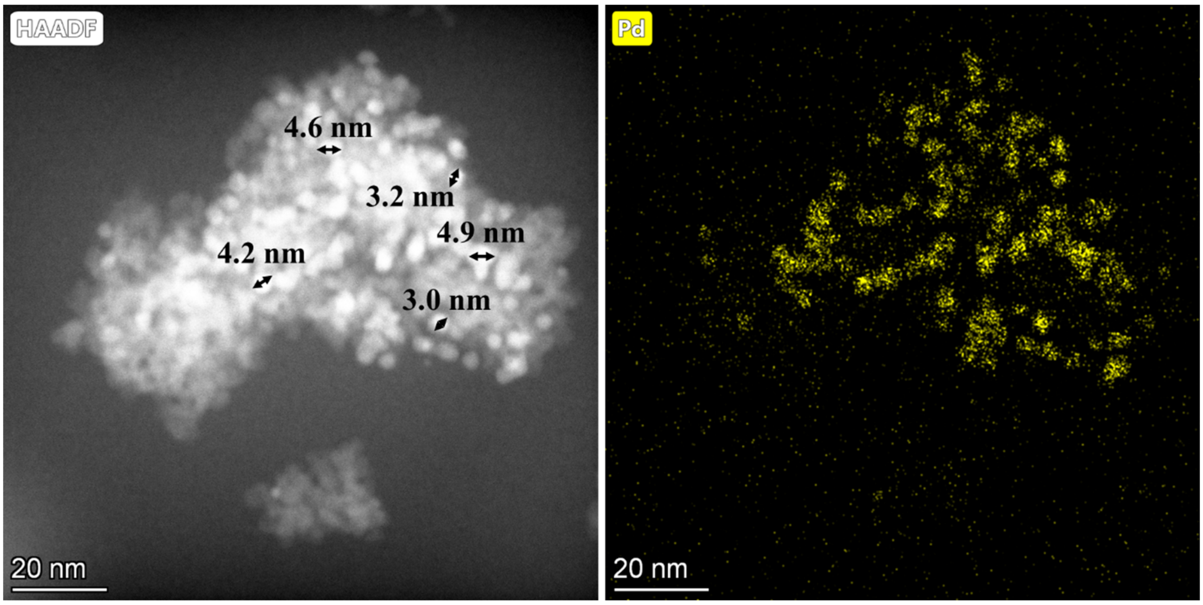

Figure S9: HAADF picture and element mapping of the $Pd/NiFe_2O_4-NH_2$ catalyst
